# Data for comparison of computed tomography angiography and dynamic myocardial perfusion to detect significant stenosis by coronary angiography

**DOI:** 10.1016/j.dib.2018.10.109

**Published:** 2018-10-26

**Authors:** Nobuo Tomizawa, Shengpu Chou, Yusuke Fujino, Masaru Kamitani, Kodai Yamamoto, Shinichi Inoh, Takeshi Nojo, Kanako K. Kumamaru, Shigeki Aoki, Sunao Nakamura

**Affiliations:** a Department of Radiology, New Tokyo Hospital, Chiba, Japan; b Department of Radiology, Juntendo University School of Medicine, Tokyo, Japan; c Department of Diabetes, New Tokyo Hospital, Chiba, Japan; d Department of Diabetes, Kashiwa Kousei General Hospital, Chiba, Japan; e Department of Cardiology, New Tokyo Hospital, Chiba, Japan

## Abstract

This article describes data related to a research article titled “Feasibility of dynamic myocardial CT perfusion using single-source 64-row CT”, Tomizawa et al. (in press) [1]. Decrease in the myocardial blood flow could be calculated by performing stress dynamic computed tomography perfusion. This article compares the receiver operating characteristics curve of computed tomography angiography and dynamic myocardial perfusion to diagnose significant stenosis by invasive coronary angiography.

**Specifications table**

**Table:** 

Subject area	*Radiology*
More specific subject area	*Cardiac imaging*
Type of data	*Figure*
How data was acquired	*Single-source 64-row CT (Somatom Definition AS+, Siemens Healthineers, Forchheim, Germany) and post processing workstation (Synapse Vincent Ver 5.1; Fujifilm Medical, Tokyo, Japan)*
Data format	*Analyzed*
Experimental factors	*CT angiography and dynamic CT perfusion was used to diagnose significant stenosis by invasive coronary angiography.*
Experimental features	*Calculating the myocardial blood flow by dynamic CT perfusion improved the diagnostic performance to detect significant stenosis than CT angiography alone.*
Data source location	*Matsudo, Chiba, Japan*
Data accessibility	*Data are within this article*

**Value of the data**

• These data provide the improvement of diagnostic performance to detect significant stenosis using dynamic CT perfusion than coronary CT angiography alone.
• These data are important because the diagnostic performance of dynamic CT perfusion using a single-source 64-row CT has not been described in the existing literature.
• These data would enable conventional 64-row CT to perform dynamic CT perfusion and allow ischemia analysis worldwide, especially when nuclear imaging, MRI or advanced CT scanners are not available.

## 1 Data

The receiver operating characteristics curves to compare the diagnostic performance of coronary computed tomography (CT) angiography alone, coronary CT angiography + visual CT perfusion, and coronary CT angiography + quantitative perfusion ratio (QPR) to predict significant stenosis by invasive coronary angiography are shown. The data were obtained from 44 patients who underwent coronary angiography after dynamic CT perfusion exam.

## 2 Experimental design, materials and methods

**Fig. 1:**
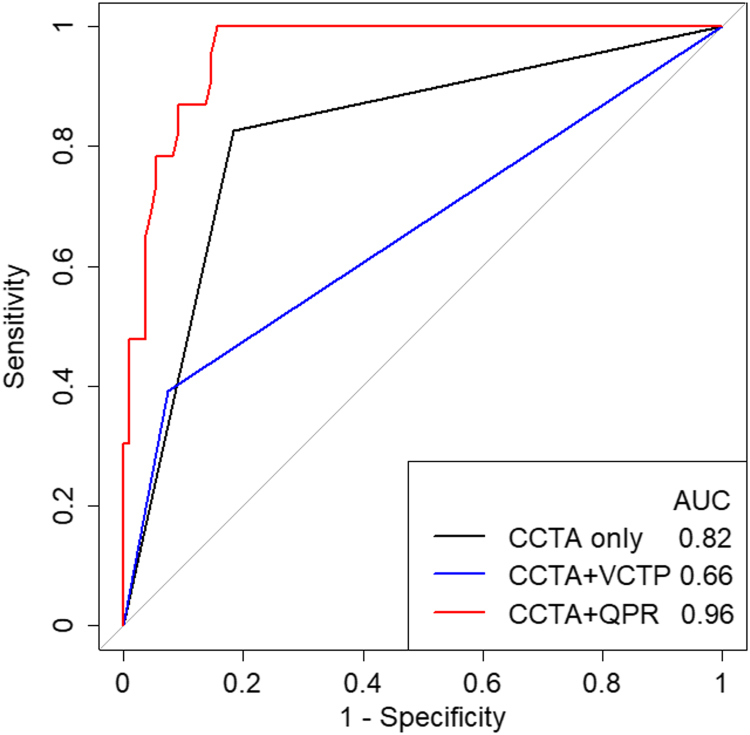
The area under the curve including QPR for analysis (red line, 0.96, 95%CI, 0.93—0.99) was significantly larger (p <0.01) than that of CCTA alone (black line, 0.82, 95%CI, 0.73—0.91) and CCTA + visual CTP (blue line, 0.66, 95%CI, 0.55—0.76). CCTA, coronary computed tomography angiography; CI, confidence interval; QCA, quantitative coronary analysis; QPR, quantitative perfusion ratio; VCTP, visual computed tomography perfusion.

A total of 44 patients who underwent subsequent coronary angiography because significant stenosis was suspected by dynamic CT perfusion exam. All patients underwent cardiac CT using a single-source CT (Somatom Definition AS+; Siemens Healthineers, Forchheim, Germany) with a collimation of 64×0.6 mm and flying-focal spot, resulting in 128 slices. The patients underwent dynamic CT perfusion under adenosine triphosphate (Adetphos; Kowa Company, Tokyo, Japan) injection at 0.14 mg/kg/min, followed by rest coronary CT angiography. Half-reconstruction images were acquired with a slice thickness of 0.75 mm and an increment of 0.4 mm using a cardiac kernel (I36f) with sinogram-affirmed iterative reconstruction strength 2 (Fig. 1).

Significant stenosis by coronary CT was defined as ≥50% stenosis. Myocardial blood flow (MBF) was calculated using a software in the workstation (Perfusion analysis; Fujifilm Medical, Tokyo, Japan). QPR of the stenosis related territory was calculated, which was defined as the MBF of the stenosis related territory divided by the remote MBF. Visual ischemia assessment was also performed. A vessel with >40% stenosis was deemed significant when decrease of MBF (QPR ≤0.96) or stress perfusion defect was not accompanied with rest perfusion defect. These data were compared to diagnose significant stenosis (≥50%) by quantitative coronary analysis (QCA; Pie Medical Imaging, Maastricht, Netherlands).

Statistical analysis was performed by R (The R Foundation for Statistical Computing).
