# Supplementary material for: Data for comparison of computed tomography angiography and dynamic myocardial perfusion to detect significant stenosis by coronary angiography
Source: Data Brief. 2018 Oct 26;21:953–5. doi: 10.1016/j.dib.2018.10.109 (PMC6222174; doi:10.1016/j.dib.2018.10.109)
Supplement: Supplementary file 1 — Supplementary material [file mmc1.docx]

Conflict of Interest Form

This manuscript was supported in part by JSPS KAKENHI Grant Number 18K15605.

The authors have no other conflict of interest regarding this article.

List of authors: Nobuo Tomizawa, Shengpu Chou, Yusuke Fujino, Masaru Kamitani, Kodai Yamamoto, Shinichi Inoh, Takeshi Nojo, Kanako K Kumamaru, Shigeki Aoki, Sunao Nakamura
